# Efficacy of post-procedural oral hydration volume on risk of contrast-induced acute kidney injury following primary percutaneous coronary intervention: study protocol for a randomized controlled trial

**DOI:** 10.1186/s13063-019-3413-5

**Published:** 2019-05-27

**Authors:** Feier Song, Guoli Sun, Jin Liu, Ji-yan Chen, Yibo He, Liwei Liu, Yong Liu, the RESCIND group

**Affiliations:** 1 Department of Cardiology, Guangdong Cardiovascular Institute, Guangdong Provincial Key Laboratory of Coronary Heart Disease, Guangdong Provincial People’s Hospital, Guangdong Academy of Medical Sciences, Guangzhou, 510080 Guangdong People’s Republic of China; 2 Guangdong Provincial People’s Hospital, School of Medicine, South China University of Technology, Guangzhou, People’s Republic of China; 3 Department of Cardiology, Guangdong Cardiovascular Institute, Guangdong Provincial Key Laboratory of Coronary Heart Disease, Guangdong Provincial People’s Hospital, Guangdong Academy of Medical Sciences, Guangzhou, 510080 People’s Republic of China; 4 The Second School of Clinical Medicine, Southern Medical University, Guangzhou, People’s Republic of China

**Keywords:** Primary percutaneous coronary intervention, Contrast-induced acute kidney injury, Oral hydration, ST-elevation myocardial infarction, Contrast media

## Abstract

**Background:**

Contrast-induced acute kidney injury (CI-AKI) contributes toward unfavorable clinical outcomes. Oral hydration with water is inexpensive and it may be effective in the prevention of CI-AKI, but its efficacy among patients undergoing primary percutaneous coronary intervention (PCI) remains unknown.

**Methods/design:**

Our study is a secondary analysis on the database from the ATTEMPT study. We enrolled ST-elevation myocardial infarction (STEMI) patients undergoing primary PCI. Eligible patients received peri-procedural aggressive (left ventricular end-diastolic pressure-guided) or routine (≤ 500 mL) intravenous hydration with an isotonic solution (0.9% NaCl) with randomization. The primary endpoint was CI-AKI, defined as a > 25% or 0.5 mg/dL increase in serum creatinine from baseline during the first 48–72 h post-procedurally. All patients drank unrestricted amounts of fluids freely, the volume of which was recorded until 24 h following primary PCI. Oral hydration volume/weight (OHV/W) ratios were calculated. The association between post-procedural oral hydration (quartiles) and CI-AKI was assessed using multivariable analysis controlling for confounders, including intravenous hydration strategies.

**Discussion:**

Our study determined the effects of post-procedural oral hydration on CI-AKI following primary PCI, which is a potential strategy for CI-AKI prevention among patients with STEMI at very high risk.

**Trial registration:**

ClinicalTrials.gov, NCT02067195. Registered on 21 February 2014.

**Electronic supplementary material:**

The online version of this article (10.1186/s13063-019-3413-5) contains supplementary material, which is available to authorized users.

## Background

Patients with ST-elevation myocardial infarction (STEMI) have a higher risk of contrast-induced acute kidney injury (CI-AKI) [1, 2]. CI-AKI is a potentially serious complication of angiographic procedures and can constitute up to 10% of hospital-acquired acute kidney injury [3]. Severe acute kidney injury requires renal replacement therapy and results in increased morbidity and mortality, prolonged hospitalization, and overall increased healthcare costs [4–6]. Of concern, 14.5% of the patients undergoing cardiac catheterization develop CI-AKI; reports have shown a 50% incidence in high-risk patients [7, 8]. CI-AKI is associated with poorer outcomes, even in STEMI patients without impaired left ventricular ejection fraction (LVEF) and with normal renal function [9, 10].

According to the 2018 ESC/EACTS guidelines on myocardial revascularization, prophylactic hydration is recommended in high-risk patients [11]. Previous meta-analyses focusing on different types of procedures (cardiac catheterization, enhanced computed tomography scans, angiography for peripheral vascular disease, and so on) have suggested that oral hydration may be as effective as intravenous hydration in preventing CI-AKI [12, 13]. Conflicting results have arisen regarding whether oral hydration prevents CI-AKI. Previous studies have been conducted on relatively low-risk patients, including those subjects undergoing intravenous radiographic procedures or elective percutaneous coronary intervention (PCI). Optimal evidence for an oral hydration strategy has not been well established in a high-risk population such as STEMI patients undergoing primary PCI.

Given the data, we designed this study to evaluate the efficacy of post-procedural oral hydration for CI-AKI prevention in high-risk patients with STEMI undergoing primary PCI.

## Methods

### Study design and population

This is a secondary analysis of the Aggressive Hydration in Patients with ST-Elevation Myocardial Infarction undergoing Primary Percutaneous Coronary Intervention to Prevent Contrast-induced Nephropathy, the First Study for Reduction of Contrast-induced Nephropathy following Cardiac Catheterization (ATTEMPT RESCIND-1) study. Patients aged ≥ 18 years with STEMI undergoing primary PCI who provided written informed consent are included from 15 academic medical centers in China. Patients are excluded for the following reasons: 1) contrast medium administration within the previous 7 days; 2) end-stage renal failure or renal transplantation; 3) inferior and/or right ventricle myocardial infarction combined with hypotension (defined as systolic pressure ≤ 90 mmHg) on admission; 4) pre-procedural renal insufficiency (history of chronic kidney disease or estimated glomerular filtration rate ≤ 60 mL/min/1.73 m^2^ calculated using the level-modified Modification of Diet in Renal Disease formula); 5) cardiogenic shock or New York Heart Association functional classification IV; 6) acute kidney injury defined as an absolute serum creatinine increase of 0.5 mg/dL from baseline obtained in the previous 24 h; 7) lactation; 8) pregnancy; 9) malignant tumor or life expectancy < 1 year; 10) allergy to contrast; 11) peri-procedural administration of nonsteroidal anti-inflammatory drugs, aminoglycosides, cyclosporine, or cisplatin in the previous 48 h or during the study period; 12) planned renal catheterization; or 13) heart valve surgery [14].

### Study protocol

All eligible participants will receive peri-procedural intravenous hydration of different regimens. Oral hydration will begin at an unlimited rate until 24 h after the coronary procedure. By monitoring the fluid status, the rate and duration of oral intake, as well as the use of diuretics, are based on the clinical evaluation for heart function and at the discretion of the cardiologists.

Additionally, oral hydration volume/weight (OHV/W) ratios will be calculated. To determine the efficacy of oral hydration, we will collect data on the type of water taken, the volume per hour of water (mL), the duration in the 24 h after the procedure, and any use of diuretics. Additionally, the urine output per hour of the patients will be observed. Hemodynamic data will be obtained in various clinical settings to assess volume status and guide medical therapy, including the administration of intravenous fluids.

Follow-up events will be carefully monitored and recorded by trained investigators through office visits and telephone interviews at 3, 6, 12, 18, and 24 months after primary PCI. All data will be collected using standardized electronic case report forms. At the time of data entry, the data management team of Guangdong General Hospital will perform consistency checks and issue electronic data clarification forms to follow-up on discrepant data.

**Fig. 1:**
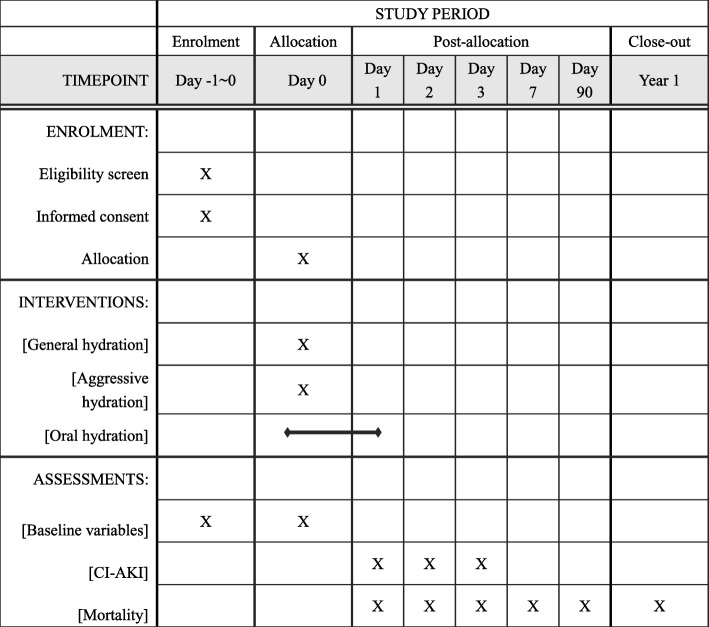
The schedule of enrolment, interventions, and assessments. CI-AKI contrast-induced acute kidney injury

Data collected at enrolment will include baseline demographics, diagnosis, medical histories, clinical and laboratory parameters, concomitant drug treatment, and physical examination findings. Confounders such as age, sex, estimated glomerular filtration rate, LVEF, the use of an intra-aortic balloon pump, chronic heart failure, anemia, diabetes mellitus, and intravenous hydration will be measured to develop the logistic regression models. The primary outcome measure is CI-AKI, defined as a > 25% or 0.5 mg/dL increase in serum creatinine from baseline during the first 48–72 h post-procedure [15]. Secondary endpoints are the different definitions of CI-AKI, as well as persistent renal damage, major adverse cardiovascular events, major post-procedure in-hospital adverse clinical events, total hospitalization costs, and length of stay (Fig. 1).

All data from the study will be collected following approval by the corresponding ethics committees of the participating centers. An independent data monitoring committee will be responsible for the review of the ongoing safety of patients enrolled in the study (Additional file 1).

### Statistical analysis

Baseline patient characteristics and information will be described as means ± standard deviation or medians and interquartile ranges of descriptive statistics. Categorical variables will be presented as frequencies and percentages. OHV/W ratios will be calculated and grouped according to the interquartile range. For continuous variables, one-way analysis of variance (ANOVA) will be conducted for normally distributed data, and the Kruskal–Wallis test used for non-normal distributions (presented as the median and interquartile range). Pearson’s chi-square test or Fisher’s exact test will be used, as appropriate, for categorical data.

The odds ratios of CI-AKI for quartiles with different OHV/W ratios (cut-off values determined by the quartiles) will be estimated using univariate and multivariate logistic regression analyses. Multivariable logistic regression models have been developed to adjust for clinical characteristics (e.g., age, sex, estimated glomerular filtration rate, LVEF, the use of an intra-aortic balloon pump, chronic heart failure, anemia, diabetes mellitus, intravenous hydration), and the Mehran risk score will be calculated [16]. All variables that are univariately associated with this outcome measure will be entered as possible predictors in a multivariable logistic regression analysis. A two-tailed *p* value < 0.05 will be considered as statistically significant. The power calculation was based on our previous findings [17], and a CI-AKI incidence of 23% was estimated for the inadequate oral hydration group, while 11.5% was assumed for the adequate oral hydration group (50% relative reduction). Using a two-sided chi-square test, a significance level of 0.05, and a sample size of 280 each group, the power of the tests is 95.44%.

All statistical analyses will be performed using SAS version 9.4 or later (SAS Institute, Cary, NC, USA) and R software (version 3.1.2; R Foundation for Statistical Computing, Vienna, Austria).

## Discussion

Patients with STEMI are likely to present with hypotension or even shock, and it is often impossible to start renal prophylactic therapy, which is associated with an increased risk of CI-AKI [18]. Despite a considerable number of risk factors, including reduced LVEF, renal dysfunction, and diabetes mellitus in patients needing adequate hydration [19, 20], cardiologists in previous studies were primarily concerned with rapid revascularization for occluded culprit arteries instead of adequate pre-procedural hydration to prevent CI-AKI; this was related to a lack of information regarding baseline renal function and related medical history. Therefore, post-procedural oral hydration might be more suitable for CI-AKI prevention in patients with STEMI.

The first study, published in 1998, reported that oral hydration was as effective as intravenous hydration [21]. Two other studies applied oral hydration to the entire group with the aim of determining the incidence of CI-AKI [22] and comparing the efficacy of different oral volumes [23] where the incidence of CI-AKI could be reduced by oral hydration. The latest three studies reported similar results [24–26]. Four meta-analyses have been published so far, which included between four and eight randomized controlled trials (RCTs) [13, 27–29]. These studies also demonstrated no significant difference between oral fluid hydration and intravenous fluid hydration regimens in the prevention of CI-AKI, nor that the oral route was inferior. There have been heterogeneities in the reported incidence of CI-AKI among studies, due to differences in CI-AKI definition, study population, and imaging procedure (Table 1).

**Table 1:** Characteristics of randomized controlled trials with intervention of oral hydration among patients undergoing coronary angiography/percutaneous coronary intervention

Study	Year	Sample size	Procedure	Contrast agent	Kidney function of participants	Contrast-induced nephropathy definition	Intervention protocols	
							Regimen one	Regimen two
Wrobel et al. [24]	2010	102	Elective CAG/PCI	Low osmolality, non-ionic (isoverol)	CKD and diabetes mellitus	> 44.2 μmol/L (> 0.5 mg/dL) absolute increase or a > 25% relative increase in serum creatinine within 72 h of contrast exposure	Oral mineral water 1 mL/kg/h for 6–12 h before and 12 h after contrast exposure	Isotonic saline, IV, 1 mL/kg/h for 6 h before and 12 h after contrast exposure (reduced to 50% in patients with CHF)
Kong et al. [26]	2012	120	Elective CAG	Low osmolality,ionic (iopromide)	Normal renal function	> 44.2 μmol/L (> 0.5 mg/dL) absolute increase or a > 25% relative increase in serum creatinine within 48–72 h of contrast exposure	Oral 2000 mL neutral water within 24 h after and/or 500 mL water before contrast exposure	Isotonic saline, IV, 1 mL/kg/h for 12 h before and 24 h after contrast exposure
Akyuz et al. [12]	2014	225	Elective CAG	Non-ionic low osmolar iopromide, Ultravist	At least one of the high-risk factors for developing CI-AKI (advanced age, type 2 diabetes mellitus, anemia, hyperuricemia, a history of cardiac failure or systolic dysfunction), eGFR ≥ 60 mL/min)	> 44.2 μmol/L (> 0.5 mg/dL) absolute increase or a > 25% relative increase in serum creatinine within 48 h of contrast exposure	Drink neutral water as much as possible for 12 h before and 2 h after contrast exposure	Isotonic saline, IV, 1 mL/kg/h for 12 h before and 12 h after contrast exposure
Cho et al. [25]	2010	91	Elective CAG	Low osmolality, non-ionic (isoverol)	CKD (baseline creatinine at least 1.1 md/dL or eGFR < 60 mL/min)	> 44.2 μmol/L (> 0.5 mg/dL) absolute increase or a > 25% relative increase in serum creatinine within 72 h of contrast exposure	Water; 500 mL started 4 h prior and stopped 2 h prior to procedure followed by oral hydration with 600 mL of water post-procedure	Isotonic saline or sodium bicarbonate solution, IV, 3 mL/kg/h for 1 h before and 6 h after contrast exposure (for patients greater than 110 kg, infusion rates will be based on that for a 110 kg person
Angoulvant et al. [30]	2009	201	Elective CAG	Ionic low osmolar (Hexabrix)	Serum creatinine < 140 μmol/L	The change in calculated creatinine clearance in 24 h and 3 days	1000 mL isotonic saline, IV, during and oral 2000 mL tap water within 24 h after contrast exposure	Oral 2000 mL tap water within 24 h after contrast exposure
Taylor et al. [21]	1998	36	Elective cardiac catheterization	Ionic contrast media in most cases	Renal dysfunction (serum creatinine ≥ 1.4 mg/dL)	An increase in creatinine of ≥ 0.5 mg/dL within 48 h of contrast exposure	Oral 1000 mL water over 10 h before then 0.45% saline, IV, 300 mL/h during and 6 h after contrast exposure	0.45% saline, IV, 75 mL/h for 12 h before and 12 h after contrast exposure

*CAG* coronary angiography, *CHF* congestive heart failure, *CI-AKI* contrast-induced acute kidney injury, *CKD* chronic kidney disease, *eGFR* estimated glomerular filtration rate, *IV* intravenous, *PCI* percutaneous coronary intervention

The above-mentioned studies were conducted on relatively low-risk patients, including those subjects undergoing intravenous radiographic procedures. The frequencies of risk factors were merely reported, and some RCTs excluded patients with chronic kidney disease, congestive heart failure, or systolic dysfunction, with a lower proportion of diabetic patients. Moreover, the oral hydration protocol varied greatly, with no two trials having a similar oral regimen.

It is reported that the incidence is < 2% in the general population but is up to 20–30% in high-risk populations with congestive heart failure, chronic kidney disease, diabetes mellitus, and anemia [3].

For in-patient settings or individuals who require emergent coronary angiography or radiological procedures with contrast exposures, intravenous hydration has been studied and used as first-line treatment for the prevention of CI-AKI [11]. However, there is no consensus regarding the mode of administration. In modern medicine, with an evolving number of diagnostic studies that depend on iodinated contrast along with an increasing number of complex high-risk patients, the costs of hospitalizations and nursing care are growing. Previous hydration strategies have not been investigated in STEMI patients. Therefore, oral hydration, which is considered safe and effective in low-risk patients, should be investigated in patients with STEMI undergoing primary PCI.

### Limitations

Our current secondary analysis is subject to the following limitations. First, it was less sensitive than defined as a > 0.5 mg/dL increase in serum creatinine, because it recognized less selectively those patients with a higher risk of mortality and morbidity. Second, according to the ATTEMPT study, participants received either aggressive or routine intravenous hydration. Hemodilution can reduce serum creatinine, and cumulative daily fluid balance (input/output) directly affects the concentration (i.e., dilution) of serum creatinine. In our study, post-procedural daily fluid balance (input/output) was recorded to estimate the change in renal function to reduce the influence of hemodilution. Third, there were the inherent limitations of an observational study, including differences in oral hydration administration protocols regarding hydration rate, time, and total volume. Fourth, the fact that post-procedure serum creatinine measurements were not random but standardized at 48 h might suggest that delayed-onset elevation of serum creatinine (> 48 h) could be overlooked. Finally, this was a secondary analysis, which is not able to conclude a causal relationship. On the basis of the above limitations, future large-sample, well-designed RCTs are required to confirm and update the findings of this secondary analysis. However, to the best of our knowledge, this is the first prospective, subanalysis to investigate the effect of oral hydration on the prevention of CI-AKI in high-risk patients undergoing primary PCI.

Oral hydration has a practical value in daily life. It is easy to administer, allows better use of hospital resources due to shorter hospital stays, does not require intravascular cannulation, is less expensive, and is more comfortable for the patient. Our study will determine the association between post-procedural oral hydration and the decreasing incidence of CI-AKI following primary PCI, as well as the risk factors among quartiles of OHV.

## Additional file

Additional file 1: SPIRIT 2013 checklist: recommended items to address in a clinical trial protocol and related documents. (DOC 121 kb)

## Abbreviations

CI-AKI: Contrast-induced acute kidney injury
LVEF: Left ventricular ejection fraction
OHV/W: Oral hydration volume/weight
PCI: Percutaneous coronary intervention
RCT: Randomized controlled trial
STEMI: ST-elevation myocardial infarction
